# Supplementary material for: Preclinical In Vivo Modeling of Pediatric Sarcoma—Promises and Limitations
Source: J Clin Med. 2021 Apr 8;10(8):1578. doi: 10.3390/jcm10081578 (PMC8069549; doi:10.3390/jcm10081578)
Supplement: Supplementary file 1 [file jcm-10-01578-s001.pdf]

Sarcoma entity	1.Genetic mutation	2. Promotor	3. Genetic strategy	4. Mode of activation	5. 2nd hit	6.Tumor developd? Histology?	7. Mouse strain	8. Penetrance (%) / Latency (Days/weeks)	9. Model details / Significance	10. Reference
**1. UPS/ Undifferentiated pleomorphic sarcoma**	Gli2A (constitutively active)	CAG, PCP2-Cre	Cag-LSL-Gli2A (Germ line transmission)	Pairing with PCP2-Cre	Pairing with SmoM2 embryonically lethal	Yes, small round cell (Ewing-like features)	Mixed C57BL/6/129Sv	98%/ 8 weeks	Gli-2induces UPS with Ewing-like-featuresby expression of EWS-ETS target genes, inlcuding Nkx2.2.	Fleming et al, Molecular Cancer Research, 2019
	KRAS^{G12D}	Rosa-26	R26^{CreER}; p53^{flox}; K^{FRT}	*P53 inactivation by systemic Tamoxifen (i.p.),local Kras activation by i.m. injection of Adeno-FlpO (Flipase)*	*P53 inactivation, Cardiotoxin injection i.m.*	Yes, UPS	Mixed C57BL/6/129Sv	*100%/8weeks (simultaneous P53 deletion and KRAS activation);8%/11 weeks (KRAS expression,3 weeks later P53 deletion);90%/6weeks (KRAS expression,3 weeks later P53 deletion,CTX treatment at day 10)*	*Muscle injury cooperates with P53 inactivation for sarcomagenesis. P53$^{-/-}$ plus muscle-injury-mediated UPS show chromosomal amplifications including YAP1/ MET.*	Van Mater et al, J CI Insight, 2018
	KRAS^{G12D}	Rosa-26	KRAS$^{LSL-G12D/+}$– ROSA26$^{LSL-Cas9eGFP}$–mice	I.m. injection of Adeno-Cre or in situ electroporation (EPO) of PX330-P-Cre plasmid	*P53 inactivation via CRISPR*	Yes, UPS (15% myogenic, 85% non-myogenic)	Mixed C57BL/6/129Sv	Ad-P-Cre:100%/10 weeks; EPO with Cas9 plasmid: 80%/11 weeks; EPOwith endogenous Cas9: 100%/7weeks	In vivo electroporation can be as effective as lentiviral delivery for conditional tumor induction.	Huang et al, Nature Communications, 2017
	P53 inactivation or hotspot mutation	–	*Conditional delection of P53$^{Lox/Lox}$ and Pten$^{Lox/Lox}$*	Local injection of Ad5-CMVE-Cre (Adenovirus) i.m. or s.c.	–	Yes, 93% UPS; 7% pleomorphic RMS	C57BL/6	100%/10weeks	First Pten-inactivated UPS model.	Buchakjian et al, PlosOne, 2017
	P53 and Pten inactivation	SFFV	*Hindlimb skeletal myoblasts isolated from P53$^{-/-}$P0 pups, lentivirally transduced in vitro with KRAS^{G12D}, transplanted into hindlimb of P53$^{-/+}$mice*	Constitutive expression	*P53$^{-/-}$*	Yes, High grade sarcoma with myofibroblastic differentiation	C57BL/6	85%/ 5 weeks	*Mutant KRAS and P53 inactivation cooperate in myoblasts for sarcomagenesis.*	McKinnon et al, Oncotarget, 2015
	KRAS^{G12D}	Rosa-26	*Pairing of LSL-KRAS$^{G12D/+}$P53$^{-/-}$–mice with 2 different Tamoxifen-inducible Cre lines: Pax7-CreER (Quiescent & activated satellite cells, myoblasts) & MyoD-CreER (activated MyoD+satellite cells and progenitors)*	Systemic tamoxifen administration	*P53$^{-/-}$, Cardiotoxin injection i.m.*	Yes, spectrum of myogenic and non myogegnic sarcoma: Myf7-CreER:62%UPS,24% eRMS, rest RMS (74% myogenic,26% non-myogenic); MyoD-CreER:100%UPS (60% myogenic,40% non-myogenic);	Mixed C57BL/6/129Sv	Myf7-CreER: 100%/ 6 weeks, MyoD-CreER: 100% /21 weeks	RMS and UPS have distinct and overlapping cells of origin within the muscle lineage. Pax7+MyoD+ quiescent satellite cells can be a cell of origin for RMS, Pax7+MyoD+cells of origin for UPS.	Blum et al, Cell Reports, 2013
	KRAS^{G12V}	Cyp1A1	Systemic overexpression/loss (germ line)	Crossing with Ah-Cremice (sceletal muscle) for systemic activation	*P53$^{+/R172H}$, P53$^{flox/R172}$, P53$^{flox/flox}$, P53$^{flox/+}$*	Yes, UPS	Mixed C57BL/6/129Sv	P53$^{+/R172H}$: 88%/10 weeks; P53$^{flox/R172}$: 100%/7 weeks; P53$^{flox/flox}$: 94%/7 weeks; P53$^{flox/+}$: 7%/15 weeks;	*Mutant P53 (P53^{R172H}) is an even more potent activator of tumorigenesis in Ras-drivenUPS than loss of P53. Low rate spontenous metastasis (13%) was only observed in mutant P53, but not loss of P53 group.*	Doyle et al, J ournal of Pathology, 2010
	KRAS^{G12D}	Rosa-26	Conditional KRAS^{G12D} overexpression	Local injection of Ad-Cre (adenovirus) into leg and uterus	*P53^{Flox}, Cdkn2a^{Flox}*	Yes, High grade sarcoma with myofibroblastic differentiation	C57BL/6	>90%/13 weeks	*KRAS-driven sarcomagenesis cooperates with Cdkn2a inactivation, but not with inactivation of Bak1 and Bax. Foxm1 expression facilitates UPS metastasis.*	Kirsch et al, Nature Medicine, 2007; Mito et al, Plos ONE, 2009;
	KRAS^{G12D}	Ryr2	Conditional KRAS^{G12V} overexpression	Cre vector pCAGnlCre delivered via electroporation of the gastrocnemius muscle of 8-10week old mice	*P53$^{-/-}$& P53$^{+/-}$*	Yes, pleomorphic RMS (Myogenin, MyoD expression not tested)	Mixed C57BL/10/129Sv	100%/6 weeks (P53$^{-/-}$) & 40%/11 weeks (P53$^{+/-}$)	*Cooperation of oncogenic RAS and P53 inactivation leads to very efficient pleomorphic sarcoma development.*	Tsumura et al, Oncogene, 2006
	P53 inactivation or hotspot mutation	–	Embryonic stem cell alteration, crossing of mice	Constitutive (germline)	–	Yes, various entities, especially lymphoma and sarcoma (typically UPS, more rarely also OS, RMS and Angiosarcoma)	Mixed C57BL/6/129Sv	10-50%/variable, typically many months	*First in vivo models for P53 inactivation, including typical mutations for Li-Fraumeni tumor predisposition syndrome.*	Donehower et al, Nature, 1992; J acks et al, Current Biology, 1994; Lang et al, Cell, 2004; Olive et al, Cell, 2004;
**2. eRMS/ Embryonal/ Fusion-negative rhabdomyo-sarcoma & pleomorphic RMS**	*Dystrophin (MDX/MtR mouse)*	–	Crossing of mice, additionally Barium chloride injection to induce muscle damage and regeneration	Constitutive (germline)	*P53$^{-/+}$ & P53$^{-/-}$ background*	Yes, RMS not further specified	C57BL/6	*MDX/mTR +P53$^{-/-}$: 100%/17 weeks; MDX/mTR +P53$^{-/+}$: 95%/35 weeks; MDX/mTR +P53$^{+/+}$: 10%/56 weeks;*	Duchenne muscular dystrophy severity facilitates RMS development. Muscle stem cells acquire RMS-like gene signature even before transformation.	Boscolo Sesillo et al, Cell Reports, 2019
	Mdm2-ALT1 (Splice variant 1)	CAG (CMV-enhancer beta-actin promotor), SOX2-Cre, also Cd19 Cre for lymphoma development	Conditional alleles activated by crossing to Cre line	Constitutive (germline)	*P53$^{+/-}$ background*	*Yes, eRMS in 50% of Mdm2-ALT1$^{-/+}$ and 0% Mdm2-ALT1$^{-/-}$, Rest lymphoma, osteosarcoma hemangiosarcoma, terratoma*	C57BL/6	*Mdm2-ALT1$^{-/+}$+P53$^{-/-}$: 100%/20weeks (50% of which are eRMS); Mdm2-ALT1$^{-/-}$+P53$^{-/-}$: 100%/27weeks (most of which are lymphoma)*	*Mdm2-ALT1 increases P53-loss-mediated eRMS tumorigenesis*	Comiskey et al, Oncogene, 2018
	hYAP1^{S127A}	Col1a, Cre lines: Pax7-cre/ERT2, Myf5-Cre, Myod1-iCre;	*Rosa26-LsL-Col1a-rtTA-TetO-YAP1^{S127A} mice paired with different Cre lines*	Constitutive or by systemic Tamoxifen administration	Cardiotoxin and Bariumchloride i.m.	Yes, eRMS	Not reported	4-8weeks, penetrance not reported	*YAP1 hyperactivity in activated, but not quiescent satellite cells, induces eRMS.*	Tremblay et al, Cancer Cell, 2014; Slemmons et al, PlosOne, 2015;
	SmoM2 (Constitutively active)	Rosa26, Cre lines: aP2-Cre, MCK-Cre, Myog-Cre, Myf5-Cre;	Conditional alleles activated by different Cre lines via mouse crossing	Constitutive (germline)	*Cdkn2a inactivation*	Yes, eRMS	Mixed	80%/6weeks for adipocyte-specific aP2-Cre; 100%/4 weeks (+Cdkn2a);	SHH activation in adipcyte progenitors can efficiently induce eRMS, particularly in cooperation with loss of Cdkn2a.	Hatley et al, Cancer Cell, 2013
	Her-2/neu	MMTV-LTR	Systemic overexpression (apart from mammary gland), Crossing of mice	Constitutive (germline)	*P53$^{-/+}$*	Yes, genitorunary eRMS, only in males	Balb/c	Close to 100%/17 weeks	*Igf2, p19Arf and p21Cip1 are upregulated in preneoplastic tissue.*	Ianzano et al, Oncotarget, 2013
	Dystrophin (MDX mouse)	–	Crossing of mice, plus Cardiotoxin (CTX) injection to induce muscle damage and regeneration;	Constitutive (germline)	*P53$^{-/-}$& P53$^{+/-}$ background*	Yes, eRMS	Mixed C57BL/10/129Sv	*Mdx/P53$^{+/+}$: 9%/43 weeks; Mdx/P53$^{+/-}$: 60%/26 weeks; Mdx/P53$^{-/-}$: 90%/17 weeks; Mdx/P53$^{-/-}$ plus CTX: 100%/13weeks; Mdx/P53$^{+/+}$ plus CTX: 0%; P53$^{-/-}$ alone: 20%/26weeks.*	P53 inactivation acclerates eRMS induction in dystrophin-inactivated mice. Muscle damage and regeneration further increases efficiency.	Camboni et al, J ournal of Pathology, 2012
	P53, Ptch and Rb1	Pax7CreER, MCre, Myf5Cre, Myf6Cre;	Conditional alleles activated by 4 different Cre lines via mouse crossing	Constitutive and via Tamoxifen (Pax7CreER)	*All tumors P53 inactivated, some also Ptch-, some also Rb1-inactivated*	Yes, eRMS, but also UPS, OS and others depending on driver and Cre line	Mixed	Variable, depending on driver and Cre line	eRMS and UPS lie in a continuum (Satellite cells predisposed towards UPS, maturing myoblasts towards eRMS).	Rubin et al, Cancer Cell, 2011
	Dystrophin (MDX mouse) & Alpha-Sarcoglycan (Sgca)	–	Crossing to hetero-and homo-zygosity of either gene, crossing with mice inactivated for 2nd hits	Constitutive (germline)	*Galgt1, Galgt2, Cmah*	Yes, eRMS	Mixed C57BL/6 & C57BL/10	*9% for mutated Dystrophin, 4% for Sgca$^{-/-}$/73 weeks*	*Muscle dystrophy-related mutations in Dystrophin and Sgca can both lead to RMS in aged mice. Tumors exhibit Mdm2 & P53 amplification with cancer-associated P53 missense mutations.*	Fernandez et al, American J ournal of Pathology, 2010
	Ptch	EIIA-Cre and Rosa26-CreERT2	*Ptch$^{+/flox}$ & Ptch$^{flox/flox}$*	Systemic tamoxifen administration embryonically and postnatally	–	*Yes, RMS and Medulloblastoma upon neonatal (Ptch$^{+/flox}$), basal cell carcinoma upon postnatal Ptch inactivation (Ptch$^{flox/flox}$)*	Mixed Balb/c and others	*20% RMS (not specified, probably eRMS-like) & 9% medulloblatoma/21 weeks, but only upon neonatal induction (Patch$^{+/flox}$)*	*Prenatally, but not postnatally induced Ptch heterozygosity resulted in the formation of RMS, accompanied by the silencing of the remaining wild-type Ptch allele. Homozygous Ptch loss was embryonically lethal, but led to basal cell carcinoma (no RMS) when induced postnatally.*	Zibat et al, Cancer Cell, 2009 et al, PlosOne, 2017
	P53 and Rb1 inactivation	SM22alpha (Specificity for smooth muscle and embryonic cardiac muscle)	Microinjection of embryos with SV40-Tumorantigen (TAg) to target P53 and RB1	Constitutive (germline)	–	Yes, cardiac RMS, not further specified, also smooth muscle proliferation in vessels	FVB/N	12 weeks (8 mice in total, penetrance not reported)	First RMS GEMM of cardiac origin.	Köbbert et al, J ournal of Thoracic and Cardiovascular Surgery, 2008
	Dystrophin (MDX mouse)	–	*MDX mouse model of Duchenne muscular dystrophy (spontaneous point mutation in exon 23 of the Dystrophin gene)*	Constitutive (germline)	–	Yes, late in life aRMS-like (fusion gene presence not analyzed, probably rather eRMS)	C57BL/10	6%/87 weeks	Mice of MDX model of Duchenne muscular dystrophy can develop RMS late in life.	Chamberlain et al, FASEB J ournal, 2007
	Sufu$^{+/-}$ (Suppressor of fused)	–	Systemic Gene trapping	Constitutive (germline)	*P53$^{-/-}$ background*	Yes, eRMS, medulloblastoma	Mixed C57BL/6 & CD1	9% /21 weeks for RMS, 58% medulloblatoma, 15% lymphoma (probably due to P53 loss alone)	*Sufu$^{-/+}$ is tumorigenic (including eRMS), but only on P53$^{-/-}$ background. Sufu$^{-/-}$ embryonically lethal. Sufu mutations not as tumorigenic as Ptch mutations.*	Lee et al, Oncogene, 2007
	SmoM2 (Constitutively active), Ptch	Rosa26 for SmoM2, CAGGs for Cre-ER (systemic)	Systemic expression of SmoM2 (Consitutively active form of SMO)	Systemic tamoxifen administration	*Ptch$^{-/+}$ background*	Yes, eRMS, basal cell carcinoma, medulloblastoma, pancreatic mucinous neoplasia	Mixed 129Sv/Swiss Webster as main components	100%/5 weeks upon tamoxifen for RMS (100% for basala cell carinoma, 40% for medulloblastoma and pancreas lesions)	Smo-mediated Sonic Hedgehoc signaling can induce multifocal eRMS with high efficiency.	Mao et al, Cancer Research, 2006
	Her-2/neu	MMTV-LTR	Systemic overexpression (apart from mammary gland), Crossing of mice	Constitutive (germline)	*P53$^{+/-}$ background*	Yes, eRMS	Balb/c	100%/14 weeks	*Her-2/neu efficiently drives genitourinary eRMS in male mice on P53$^{-/-}$ background, but not in females.*	Nanni et al, Cancer Research, 2003
	FOS & P53	–	Systemic inactivation, Crossing of mice	Constitutive (germline)	–	Yes, eRMS	Mixed C57BL/6/129Sv	90%/16 weeks for homozygous double knockout, less efficient in heterozygosity	*Fos/P53 double knockout mice develop eRMS in facial and orbital regions.*	Fleischmann et al, Cancer Cell, 2003
	HGF/HF	MT-1	Systemic overexpression, Crossing of mice	Constitutive (germline)	*Ink4a/Arf inactivation*	Yes, eRMS	C57BL/6	Mixed FVB/C57BL/6	*Ink4a/Arf inactivation increases efficiency of c-Met-tumorigenicity.*	Sharp et al, Nature Medicine, 2002
	Ptch (Patch)	–	Systemic inactivation (exons 6 & 7), Crossing of mice	Constitutive (germline)	–	Yes, eRMS	Mixed C57BL/6 & CD1	9% in CD1, 2% in C57BL/6/latency na	*Germline GEMM of Gorlin syndrome with typical features and eRMS (with Gli1 and Igf2 overexpression). Ptch$^{-/-}$ embryonically lethal.*	Hahn et al, Nature Medicine, 1998
	HGF/HF	MT-1	Systemic overexpression, Crossing of mice	Constitutive (germline)	–	Yes, RMS not further specified, amelanotic melanoma, hepatic and mammary tumors	FVB/N	7% RMS/latency na	Activation of c-Met-tyrosinekinase via autocrine signaling is tumorigenic.	Takayama et al, PNAS, 1997
**3. aRMS/ Alveolar/ Fusion-positive rhabdomyo-sarcoma**	*Pax3-Foxo1*	Endogenous Pax3 locus; Cre line: Myf6Cre	Conditional alleles, targeted via Cre	Crossing of conditional mice with Cre lines	*Inactivation of Cdkn2a, Pax3 & Stk 3&4*	Yes, aRMS	Mixed C57BL/6/129Sv	*Stk3&4$^{-/-}$: 88%/16 weeks; Stk3&4$^{+/+}$: 27%/26 weeks;*	*Activated Hippo signaling increases tumorigenesis in Cdkn2a-inactivated aRMS.*	Oristian et al, Cancer Research, 2018
	Pax3-Foxo1	Endogenous Pax3 locus; Cre lines: Pax7CreER, Myf5CreER, Myf6CreER, Mcre;	*Overexpression via Cre-LoxP-conditional Pax3-Foxo1 knockin allele*	Systemic Tamoxifen administration at P30	*P53, Pax3 inactivation*	Yes, aRMS (Pax7CreER tumors showed spindle/pleomorphic morphology)	Mixed C57BL/6/129Sv	MCre: 40%/29 weeks; Myf6CreER: 100%/15 weeks; Pax7CreER: 65%/48 weeks; Myf5-Cre: Embryonically lethal except for one (tumor-bearing) mouse;	aRMS can arise from different muscle lineages, particularly efficient in maturing myoblasts.	Abraham et al, Genes and Development, 2014
	Pax3-Foxo1	Endogenous Pax3 locus; Cre lines: Myf6 (differentiateing myogenic cells);	*Systemic overexpression via Cre-LoxP-conditional Pax3-Foxo1 knockin allele (partly plus conditional Foxo1-knockout allele)*	Crossing of conditional mice with Cre lines	*Ink4a/Arf or P53 inactivation*	Yes, aRMS	Mixed C57BL/6/129Sv	*0.5%/55weeks (fusion alone); 8%/31 weeks (P53$^{+/-}$); 40%/13 weeks (P53$^{-/-}$); 0% (Ink4a/Arf$^{+/-}$); 29%/10 weeks (Ink4a/Arf$^{-/-}$);*	*Pax3-Foxo1 leads to tumorigenesis when expressed in Myf6+ cells, efficiency is increased by P53 and Cdkn2a deletion.*	Keller et al, Genes and Development, 2004 (2 articles) & Nishijo et al, Cancer Research, 2009
	Pax3-Foxo1	Endogenous Pax3 locus (PGK-, MyoD-and rat-beta-actin promotor also tried)	Pairing of mice	Constitutive (germline)	–	No	Mixed C57BL/6/NMRI	–	*Heterozygous and chimeric mice showed developmental muscle defects and died perinatally from cardiac/respiratory failure. Pax3-Foxo1 expression from PGK-, MyoD-and rat-beta-actin promotor did not yield any phenotype.*	Lagutina et al, Molecular and Cellular Bioloyg, 2002
**4. MYOD1-RMS**	No modeling attempts identified in the literature. Only one comprehensively characterized cell line model described (Schleicher et al, Cells, 2020).									
**5. OS/ Osteosarcoma**	*Rb, P53*	–	*Conditional knockdown (shP53) & conditional knockout Rb$^{flox/+}$, Rb$^{flox/flox}$*	Crossing with Osx-Cre mice	–	Yes, OS (osteoblastic subtype)	C57BL/6	100%/58 weeks	In contrast to other osteosarcoma models that used Cre:lox mediated gene deletion, osteosarcoma generated through shRNA-based knockdown of p53 results in osteoblastic OS, the most prevalent form sub-type of human osteosarcoma.	Mutsaers et al, Bone, 2013
	SV40 TAg	Og2	SV40 expression under the Og2 promoter	Crossing of mice	–	Yes, OS	FVB	100%/11-20weeks	Prkar1a is a bone tumor suppressor gene capable of directing subclass development in mouse and human OS.	Molyneux et al, The J ournal of clinical investigation, 2010
	Rb, P53	–	*Conditional inactivation of P53$^{flox/+}$, Rb$^{flox/+}$, P53$^{flox/flox}$, Rb$^{flox/flox}$*	Crossing with Prx-1-Cre mice	–	Yes, OS (and soft-tissue sarcoma with lower penetrance)	C57BL/6	92%/19 weeks	The use of Cre in Prx-positive progenitors also leads to development tumors of other mesenchymal linages at high frequencies.	Lin et al, Carcinogenesis, 2009; Calo et al, Nature, 2010
	P53	–	*Conditional inactivation of P53$^{flox/+}$, P53$^{flox/flox}$*	Crossing with Prx-1-Cre mice	–	Yes, OS (and soft-tissue sarcoma with lower penetrance)	C57BL/6	22%/96 weeks for heterozygous; 61%/50 weeks for homozygous mice;	The use of Cre in Prx positive progenitors also leads to development tumors of other mesenchymal linages at high frequencies.	Lin et al, Carcinogenesis, 2009; Calo et al, Nature, 2010
	P53	–	*Conditional inactivation of P53$^{flox/flox}$*	Crossing with Col1A1-2.3-Cre mice	–	Yes, OS	C57BL/6	85%/ 5 weeks	Deletion of P53 in committed osteoblasts via Col1a1-Cre still results in a high percentage of OS like in Prx1-cre progenitors.	Lin et al, Carcinogenesis, 2009
	Rb, P53	–	*Conditional inactivation of P53$^{flox/+}$, Rb$^{flox/+}$, P53$^{flox/flox}$, Rb$^{flox/flox}$*	Crossing with Osx-Cre mice	–	Yes, OS	Mixed C57BL/6-J /129	100%/ 23 weeks; 100%/18 weeks;	Cooperation of P53 and RB1 inactivation accelerates OS tumorigenesis.	Berman et al, PNAS, 2008; Walkley et al, Gen & Dev, 2008
	Rb, P53	–	*Conditional inactivation of P53$^{flox/flox}$*	Crossing with Osx-Cre mice	–	Yes, OS	Mixed C57BL/6-J /129	100%/ 41weeks	OS exhibited many of the features characteristic of human OS, including comparable histology, metastatic site preference, karyotypic complexity, and transcriptional profiles.	Berman et al, PNAS, 2008; Walkley et al, Gen & Dev, 2008
	Rb, P53	–	*Conditional inactivation of P53$^{flox/flox}$*	Crossing with Col1A1-3.6-Cre mice	–	Yes, OS (20% incidence of lymphoma or fibrosarcoma)	Not reported	60%/42 weeks	Mice specifically deleted for P53 in osteoblasts develop osteosarcomas, wich correlates with increased proliferation, increased expression of RUNX2, increased osteoblast maturation as a result of P53 inactivation.	Lengner et al, The J ournal of Cell Biology, 2006
	P53$^{R172H/+}$	–	*Conditional activation of P53$^{R172H/+}$ mutant*	Lox-STOP-Lox (LSL) cassette at the p53 locus; Prm-Cre (germline)	–	Yes, OS in 50% (also develop carcinomas, lymphomas and other tumors)	129/SvJ	50%/mean survival time 67 weeks	*P53 mutant mice develop tumor spectra distinct from P53$^{+/-}$ mice. In contrast to carcinomas in P53$^{R270H/+}$ mice, the most frequent tumors in the p53$^{R172H/+}$ mice were osteosarcomas.*	Lang et al, Cell, 2004; Olive et al, Cell, 2004;
	P53	–	Embryonic stem cell alteration, Constitutive (germline)	Crossing of mice (germline)	–	Yes, various entities, especially lymphoma and sarcoma (typically UPS, more rarely also OS, RMS and Angiosarcoma)	129/SvJ	4%/16-20weeks, 25%/39 weeks	The unexpected ratio of tumor formation in homozygous vs heterzygous mice is due to the early lethality seen in the homozygous mice (rapid development and higher incidence of other tumors-mostly lymphomas, and the long latency of OS).	Donehower et al, Nature, 1992; J acks et al, Current Biology, 1994;
**6. EwS/Ewing sarcoma**	A EwS-GEMM has been elusive so far despite 16 attempts by 6 different laboratories to express EWSR1-FLI1 via various conditional and tissue-specific strategies and using a plethora of different promotors (Runx2, Col1a2.3, Col1a3.6, Prx1, CAG, Nse, NEFL, Dermo1, P0, Sox9 and Osterix) by Minas/Surdez/J avaheri/Tanaka/Howarth/Kang et al, Cancer Research, 2017 Minas/Surdez/J avaheri/Tanaka/Howarth/Kang et al, Cancer Research, 2017 Minas/Surdez/J avaheri/Tanaka/Howarth/Kang et al, Cancer Research, 2017									
**7. SySo/Synovial sarcoma**	SS18-SSX1/2	Rosa26	Loxp-PGK-Neo-tPA-Loxp-SS18-SSX2/1 (human cDNA)	Conditional activation using Myf5-Cre or TAT-cre injection/pairing of mice.	*SS18-SSX2 is suficient to drive tumorigenesis in Myf5 positive progenitors. Accelarated by Pten inactivation or expression of a stabilized version of Beta-Catenin.*	Yes, SySo developed in the skeletal-muscle. Histology similar to human tumors, with both biphasic and monophasic variants.	Mixed SvJ -129/ C57BL/6	100%/12-20weeks (My5-Cre mice)	SS18-SSX1/2 allele avaiable with and without an IRES-GFP reporter. SS18-SSX1/2 allele can also be activated via a a tamoxifen-inducible CreER system or by TAT-CRE delivery (focal tumorigenesis albeit with longer latency)	Haldar et al, Cancer Cell, 2007; Haldar et al, Cancer Research, 2009; Barrott et al, Oncotarget, 2015; Barrott et al, The J ournal of Experimental medicine, 2016;
**8. MPNST/ Malignant peri-pheral nerve sheath tumor**	*Nf1, P53*	U6 for sgRNAs, CBh for Cas9	One construct with 2 linked sgRNAs vs Nf1 and P53 plus Cas9 packed into adenovirus	Targeted injection into the sciatic nerve	–	Yes, MPNST	129/SvJ	83%/14 weeks	Validation of CRISPR-technology to induce the same tumorigenesis of similar latency and molecular makeup as CreLoxP-approach.	Huang et al, Nature Communications, 2017
	Nf1, Ink4a/Arf	–	Systemic Nf1$^{flox/flox}$ & Ink4a/Arf$^{flox/flox}$, spatially controlled inactivation by Cre injection	Ad-Cre injection into the sciatic nerve or the thigh muscle	–	Yes, MPNST when injected into the sciatic nerve, 50% UPS & 50% RMS when injected into the muscle (H.E. IHC)	Not reported	100%/18 weeks for MPNST; 100%/26 weeks for RMS & UPS;	Conditional MPNST model with wildtype P53 background.	Dodd et al, Molecular Cancer Therapeutics, 2013
	Nf1, Ink4a/Arf	–	Nf1$^{+/-}$ + Ink4a/Arf$^{-/-}$ or P53$^{+/-}$; Crossing of mice;	Constitutive (germline)	–	Yes, MPNST	C57BL/Ka	Variable, dependent on combination	The tumorigenic potential of MPNST cells with Nf1$^{+/-}$ and Ink4a/Arf$^{-/-}$ is higher than Nf1$^{+/-}$ and P53$^{+/-}$, tumorigenisity depends on laminin.	Buchstaller et al, Cancer Cell, 2012
	Nf1, P53	–	Nf1$^{Flox/-}$ & previous models (conditional knockout)	Crossing with Wnt-1-Cre+/ Periostin-Cre+/ P0a-Cre+mice	–	Yes, Neurofibroma, MPNST, others	Mixed C57BL/6/129Sv	Variable, dependent on combination	Nf1$^{+/-}$, Ink4a/Arf$^{-/-}$ in Schwann, but not neuronal crest cells leads to MPNST development.	J oseph et al, Cancer Cell, 2008
	Nf1, P53	–	Nf1$^{+/-}$& P53$^{+/-}$	Constitutive (germline)	–	Yes, sarcoma, MPNST only NP-Cis animals	Mixed C57BL/6/129Sv	*100%/26 weeks for Nf1$^{+/-}$ and P53$^{+/-}$ (NP-CIS); 56 weeks for Nf1$^{+/-}$ and P53$^{+/-}$ (NP-Trans);*	*Heterozygosity of Nf1$^{+/-}$ and P53$^{+/-}$ on the same chromoseome leads to faster MPNST formation than on opposite chromosomes; Chimeras for Nf1-deletion show varying developmental defects and plexiform neurofibromas depending on degree of chimerism.*	Cichowski et al, Science, 1999
	Nf1, P53	–	Nf1$^{-/-}$& P53$^{+/-}$; Nf1$^{+/-}$& P53$^{+/-}$ (Constitutive/Germline)	Constitutive (germline)	–	Yes, 77% sarcoma (mostly MPNST, but also RMS, MTT, LMS), 14% lymphoma, 8% carcinoma, 3% neuroblastoma	Mixed C57BL/6/129Sv	*100%/17 weeks for Nf1$^{-/-}$ and P53$^{+/-}$; 22 weeks for Nf1$^{+/-}$ and P53$^{+/-}$;*	*Germ line homozygosity for Nf1 deletion leads to embryonic lethality.*	Vogel et al, Science, 1999
**9. IFS/Infantile fibrosarcoma**	No holistic GEM modeling attempt could be identified. Nonetheless, both ETV6-NTRK3 and EML4-NTRK3 tranform NIH3T3 fibroblasts and positively engraft in immunocompromised mice s.c. (SCID/NSG mice). (Wai et al, Oncogene, 2000 & Tannenbaum-Dvir et al, Cold Spring Harb. Mol. Case Studies, 2015)									
**10. MRT / Malignant rhaboid tumor**	*SmarcB1*	–	*Smarcb1$^{flox/flox}$*	Crossing with Mx-Cre (Systemic inactivation)	*P53$^{flox/flox}$*	Yes, MRT histology, negative for SNF5 expression. Mice also develop mature CD8+ T cell lymphoma	Mixed C57BL/6/129Sv	100%/11 weeks; 100%/ 3 weeks when p53 is co-inactivated	*Conditional inactivation of SmarcB1 results in highly penetrant cancer predisposition with 100% of mice developing mature CD8+T cell lymphoma or rabdoid tumors. While loss of SNF5 predisposes to aggressive cancers, it is also required for survival of virtually all nonmalignant cells in vivo.*	Roberst et al, Cancer Cell, 2002; Isakoff et al, PNAS, 2005;
	SmarcB1	–	*SmarcB1$^{+/-}$; SmarcB$^{1-/}$–;*	Constitutive (germline)	P53 co-inactivation	Yes, MRT, negative for SNF5 expression	Mixed C57BL/6/129Sv	12%/5 weeks	*Smarcb1 homozygous mice are embryonicaly letal. Heterozygous mice are predisposed to MRT. P53 (but not Cdkn2a or Rb inactivation) accelerates tumor formation.*	Roberts et al, PNAS, 2000; Guidi et al, Mol Cell Biol, 2001; Klochendler-Yeivin et al, EMBO reports, 2000; DelBove et al, Molecular carcinogenesis, 2009;
**11. CSSK/ Clear cell sarcoma of the kidney**	No modeling attempts identified in the literature.									
**12. CSS/ Clear cell sarcoma of soft tissue**	*EWSR1-ATF1 (human cDNA)*	Rosa26	Conditional expression cassette (loxP-neo-R+polyA-loxP-EWS-ATF1-IRES-GFP) under Rosa26 or Rosa26-CreER in heterozygous mice	Local injection of Tat-Cre into limbs and various other locations plus systemic Tamoxifen-mediated activation (Rosa26-CreER)	–	Yes, CSS resembling human disease	C57BL/6	100%/5weeks (100µM Tat-Cre) to 40 weeks (2µM Tat-Cre)	A variety of mouse cells are permissive to EWSR1-ATF1-transformation, embryonic expression is lethal, expression in young mice leads to stunted growth.	Straessler et al, Cancer Cell, 2013
**13. ASPS/ Alveolar soft part sarcoma**	*ASPSCR1-TFE3 human cDNA*	Rosa26	Rosa26-LSL-AT3 crossed to systemic CreER	Systemic Tamoxifen-mediated activation (Rosa26-CreER)	–	Yes, tumors recapitulated human ASPS histopathologically and in expression analysis	Mixed 129S1/SvImJ and C57BL/6	100%/22 weeks	The completely penetrant tumors were indistinguishable from human ASPS by histology and gene expression. Tumors formed preferentially in an anatomic environment highest in lactate. No concurrent genetic hits were required for tumorgenesis.	Goodwin et al, Cancer Cell, 2014
**14. SBRCT-BCOR /Small Blue round cell tumor**	No modeling attempts identified in the literature.									
**15. SBRCT-CIC /Small Blue round cell tumor**	No modeling attempts identified in the literature.									
**16. DSRCT/ Desmoplastic small round cell tumor**	No modeling attempts identified in the literature.									
**17. MC/ Mesenchymal chondrosarcoma**	No modeling attempts identified in the literature.									
**18. IMT/ Inflammatory myofibroblastic tumor**	No modeling attempts identified in the literature.									

Supplemental figure 1. Existing GEM models for pediatric sarcoma of 18 focus entities. *For each entitiy published GEM models are presented in chronological order, starting with the most current approach. A color code was applied to highlight groups of models within recurrent genetic pathways/networks: Sonic Hedgehog signaling, RAS signaling, Hippo signaling/Muscle injury/Muscular dystrophy, HER-2/Neusignaling, HGF/HF signaling. P53, Rb1 and Cdkn2a inactivation was so commonly applied that it did not receive a specific color code. For further details on individual models, see text and original articles.*
